# MOTS-c and Exercise Restore Cardiac Function by Activating of NRG1-ErbB Signaling in Diabetic Rats

**DOI:** 10.3389/fendo.2022.812032

**Published:** 2022-03-17

**Authors:** Shunchang Li, Manda Wang, Jiacheng Ma, Xiaoli Pang, Jinghan Yuan, Yanrong Pan, Yu Fu, Ismail Laher

**Affiliations:** ^1^ Institute of Sports Medicine and Health, Chengdu Sport University, Chengdu, China; ^2^ Department of Pharmacology and Therapeutics, Faculty of Medicine, University of British Columbia, Vancouver, BC, Canada

**Keywords:** MOTS-c, aerobic exercise, type 2 diabetes (T2D), myocardium, transcriptome

## Abstract

Pathologic cardiac remodeling and dysfunction are the most common complications of type 2 diabetes. Physical exercise is important in inhibiting myocardial pathologic remodeling and restoring cardiac function in diabetes. The mitochondrial-derived peptide MOTS-c has exercise-like effects by improving insulin resistance, combatting hyperglycemia, and reducing lipid accumulation. We investigated the effects and transcriptomic profiling of MOTS-c and aerobic exercise on cardiac properties in a rat model of type 2 diabetes which was induced by feeding a high fat high sugar diet combined with an injection of a low dose of streptozotocin. Both aerobic exercise and MOTS-c treatment reduced abnormalities in cardiac structure and function. Transcriptomic function enrichment analysis revealed that MOTS-c had exercise-like effects on inflammation, myocardial apoptosis, angiogenesis and endothelial cell proliferation and migration, and showed that the NRG1-ErbB4 pathway might be an important component in both MOTS-c and exercise induced attenuation of cardiac dysfunction in diabetes. Moreover, our findings suggest that MOTS-c activates NRG1-ErbB4 signaling and mimics exercise-induced cardio-protection in diabetes.

## Introduction

Type 2 Diabetes (T2D) is a global epidemic that is expected to affect over half a billion people worldwide by 2030 (1, 2). Diabetes independently increases the risk of heart failure by up to 5-fold in women and 2-fold in men (3–5). Cardiac structural remodeling and diastolic dysfunction are the main causes of heart failure and death in patients with diabetes (6–8). The pathogenesis of diabetic cardiac diastolic dysfunction is complicated, with suggested primary causes including metabolic disorders, myocardial fibrosis, microangiopathy, oxidative stress, inflammatory reaction, and defective calcium transport in cardiomyocytes (9–14).

Regular aerobic exercise in type 2 diabetes improves blood glucose levels, increases the myocardial activity of antioxidant enzymes (15, 16), and the bioavailability of nitric oxide (NO) (17–19). Aerobic exercise lowers myocardial oxidative stress, improves left ventricular contractility, and reduces disorders of glucose and lipid metabolism in diabetic rats, leading to improvements in ventricular fibrosis and diabetic cardiac diastolic dysfunction (20, 21).

Although exercise therapy is often used as an important part of the comprehensive routine treatment for type 2 diabetes, its therapeutic effects have been greatly weakened by exercise intolerance and low compliance rates (22–25). Recently discovered exercise mimetics are expected to promote or partially replace the therapeutic effects by mimicking the beneficial effects of exercise training (26–28).

The mitochondrial open-reading-frame of 12S rRNA-c (MOTS-c) is an exercise candidate mimetic (27, 29) that increases aminoimidazole carboxamide ribonucleotide (AICAR), activates 5-adenosine monophosphate activated protein kinase (AMPK) at the cellular level; MOTS-c confers some exercise-like effects by lowering blood glucose levels (29), enhances the clearance of glucose in glucose tolerance tests (30), reduces fatty acids levels and lipid accumulation in the liver of mice fed a normal diet (31). Treatment with MOTS-c increases the expression of transcriptional activator 3 and aromatic hydrocarbon receptors by reducing mitogen-activated protein kinase (MAPK) phosphorylation, enhances the bactericidal ability of macrophages, and inhibits the expression of pro-inflammatory cytokines such as interleukin IL-6 and IL-1β (32).

In this study, we used RNA-Seq technology to assess the effects of MOTS-c and aerobic exercise on the myocardial transcriptome in normal and T2D rats, analyze the differentially expressed genes (DEGs) and their functional associations, and explore the overlapping mechanisms of aerobic exercise and MOTS-c supplementation in the treatment of diabetic cardiac dysfunction. Our findings provide experimental evidence and a theoretical basis for using MOTS-c supplements to mimic the exercise-like cardiac benefits in diabetes.

## Materials and Methods

### Animal Care

6-week-old male Sprague Dawley (SD) rats were purchased from Chengdu Da-shuo Experimental Animals Co. Ltd. (Chengdu, China) and housed in standard poly-propylene cages under a 12 h/12 h light/dark cycle, at ambient temperatures of 21–23°C and a relative humidity of 40–60%. All animals were allowed free access to rat chow and water ad libitum. Experiments were approved by the Academic Committee of Chengdu Sport University (No: 2021-07).

Fifty-five animals were randomly divided into control (C, n=10) and high-fat high-sugar diet plus streptozotocin (STZ) treatment groups (D, n=45). Thirty diabetic rats were randomly divided into three groups of ten rats per group: diabetes (D), diabetes exercised (DE), and diabetes plus MOTS-c treatment (DM)

### Induction of Type 2 Diabetes

Forty-five SD rats was fed a high-fat high-sugar diet for 7 weeks, and then injected with a low does of streptozotocin (STZ, Sigma-Aldrich, St Louis, MO, 30mg/kg, i.p) (33). STZ was dissolved in sodium citrate buffer (0.1 mol/L, pH 4.4) (34). Rats in the control group were injected with a vehicle of citrate buffer (0.25 ml/kg). Normal rat chow consisted of 5% fat, 50% carbohydrate, and 23% protein, while the high-fat diet contained 67% normal pellets, 10% lard, 20% sucrose, 2% cholesterol, and 1% sodium cholate (35). Both normal and high-fat rat chow was supplied by Chengdu Da-shuo Biotech Co. Ltd. (Chengdu, China). Diabetes was confirmed within 3 days after STZ injection when the blood glucose measurements were greater than 16.7 mmol/L (36). Signs of type 2 diabetes occurred in 71% of the rats.

### Exercise and MOTS-c Injection Protocols

Aerobic exercise protocol: A motor-driven treadmill (model SA101, Jiangsu SANS Biological Technology Co., Ltd., China) was used for aerobic exercise training. Rats were acclimated to the treadmill during the first three days when the treadmill speed was set for 5 min at 8 m/min speed and then changed to 10 min at 10 m/min. The aerobic exercise protocol was adapted from the Bedford classic motion model (37). Rats in the aerobic exercise groups ran progressively faster to a speed of 20m/min for 1 h, which corresponded to approximately 50–60% of their VO_2max_. Tail stimulation with compressed air and tactile stimuli was used to prompt continuous running of rats during the aerobic exercise sessions. Aerobic exercise lasted 1h per day for 5 days a week for 8 weeks.

MOTS-c treatment protocol: Rats in the DM group were injected with MOTS-c (0.5mg/kg/day, i.p.), for 7 days/week for 8 weeks, while rats in group C were injected with the same amount of citrate buffer (0.25 ml/kg).

### Assessment of Plasma Glucose and Insulin Levels

Plasma glucose levels were estimated with a freestyle light blood glucose monitoring system (Jannsen, New Jersey, USA). Fasting insulin levels were measured in triplicate using ELISA kits for rat insulin (ImmunoWay, Plano, Texas, USA) with a plate reader SpectraMax M5 (Thermo Scientific, Waltham, Massachusetts, USA) at 450 nm. Assays followed manufacturer instructions without modifications. The body weights (BW) of rats were measured once a week.

### Calculation of HOMA-IR Indexes

An individual Homeostatic Model Assessment for Insulin Resistance (HOMA-IR) index was determined for each animal using plasma glucose and insulin levels as per the formula: HOMA-IR = Fasting blood glucose (FBG) (mmol/L) × Fasting insulin (FINS) (mU/L)/22.5 (38).

### Measurement of Cardiac Structure

Hematoxylin-eosin staining (HE): Left ventricular tissue was placed in 4% polyformaldehyde, embedded in paraffin and cut into 4-5μm thick serial sections. Slices were deparaffinized in a sectioning machine (LEICA RM 2245), treated with xylene, stained with hematoxylin-eosin and dehydrated by subsequent immersions in graded alcohol solutions, and were then sealed with neutral gum. Myocardial structure was visualized using an optical microscope (Olympus, Tokyo, Japan, BX53).

Transmission Electron Microscopy (TEM): The cardiac left ventricle was fixed in 3% glutaraldehyde, subjected to 1% tetroxide re-fixation, pyruvate dehydration, embedded in epoxy resin (Epon) 812, and cut into 50nm thin slices. Uranium acetate and lead citrate double staining was performed and tissue slices were viewed with a H-600IV transmission electron microscope.

### Measurement of Cardiac Function

Small animal echocardiography (Philips, CX50) was used to detect changes of left ventricular internal diameter during systole (LVIDs), ejection fraction (EF), fractional shortening (%FS), peak early (E-wave peak) and atrial blood flow velocities (A-wave peak), and for measuring the ratio of early to late (atrial) ventricular filling velocity (E/A ratio). For determination of cardiac structure and function, images of the short axis of the left ventricle at the mid-ventricle level and long axis images of the left ventricle were obtained and stored as digital video loops. M-mode echocardiograms were obtained from the short axis view.

### Transcriptomic Profiling

#### Construction of Gene Library

The purity, concentration and integrity of RNA samples ensured that only well characterized samples were used for transcriptome sequencing. The main steps were: (1) Oligo (DT) - loaded magnetic beads were used to enrich eukaryotic mRNA; (2) The mRNA was randomly interrupted by adding fragmentation buffer; (3) The first cDNA strand was synthesized with random hexamers using mRNA as the template. The second cDNA strand was then synthesized by adding buffer, dNTPs, RNase H and DNA polymerase I. The cDNA was purified using ampere XP beads; (4) The purified double stranded cDNA was repaired by adding a tail and connecting it to the sequencing adaptor, and the fragment size was then selected by AMPure XP beads (5); Finally, the cDNA library was obtained by PCR enrichment. After construction of the library, the effective concentration of the library (effective concentration of the library > 2nM) was accurately quantified by Q-PCR to ensure the quality of the library. After the library was qualified, the different libraries were pooled according to the target amount of offline data and sequenced using the Illumina platform.

#### Quality Control

Filtered data was generated from raw data in FASTQ format using the following filtering criteria: (1) Remove reads containing the connector; (2) Remove low-quality reads (including those of more than 10% of N (fuzzy bases); (3) Base numbers of Q ≤ 10 were removed, which accounted for more than 50% of the whole reads. We obtained high quality clean data after applying these quality control measures. HISAT2 (39) and StringTie (40) were used to compare the transcriptome data with the reference genome sequence.

#### Screening of Differentially Expressed Genes

For samples with biological duplication, edgeR (41) was used to obtain differential expression of sample gene sets between two biological conditions. edgeR was used for difference analysis for samples without biological duplication. The p-values were adjusted by applying the false discovery rate (FDR) to control false discovery rates. Genes were considered as expressed differentially if the corrected p-value was < 0.05 and fold change was ≥1.5.

Functional enrichment analysis: Gene Ontology (GO) enrichment analysis of differentially expressed genes (DEGs) was applied using Cytoscape (version 3.8.2) plug-in ClueGO (version 2.5.7). The GO terms were considered significantly enriched with an adjusted p-value < 0.05. ClueGO was also used for clustering and visualizing GO terms to create functionally organized networks with default settings.

### Quantitative Real-Time PCR (qRT-PCR)

Total RNA was modified to cDNA using the Animal Total RNA Isolation Kit (Foregene, Chengdu, China) and the 5× All-In-One MasterMix (AccuRT Genomic DNA Removal kit) (ABM, China). We followed the manufacturer’s instructions, using 10-20 mg of left ventricular apex to extract total RNA by adding buffer, DNA cleaning column and RNase free ddH_2_O, etc. A total of 2μg of RNA samples was reverse transcribed. The EvaGreen Express 2×qPCR MasterMix-No Dye (ABM, China) was used to prepare the qRT-PCR reaction mixture. RT-PCR was performed using a SLAN-96S Real-Time PCR system (Shanghai, China) with an amplification procedure of 95°C for 10 min, 40 cycles of 95°C for 10 s and 60°C for 30 s. The gene of β-actin was used to normalize the expression levels of selected genes, and the relative expression of mRNAs was analyzed using the 2−ΔΔCt method. Specific primers were designed by Sangon Biotech Co., Ltd (Chengdu, China) and presented in **Table 1**.

**Table 1:** qRT-PCR primers.

mRNA (GenBank No)	Primer	Size (bp)
NRG1(112400)	Forward: GTCGGCATCATGTGTGTGGT	102
	Reverse: CAGGTTGCTCCGTTCTGACC	
ErbB (24329)	Forward: AGGAGGTGGCTGGCTATGTTCTC	127
	Reverse: TAGTTGGACAGGACGGCTAAGGC	
β-Actin (81822)	Forward: TGTCACCAACTGGGACGATA	165
	Reverse: GGGGTGTTGAAGGTCTCAAA	

### Western Blotting

Left ventricular myocardial tissues was lysed in radio-immunoprecipitation lysis buffer (containing 1% broad spectrum protease inhibitors) for protein extraction. The tissues were vortexed for 30 min at 4°C and centrifuged at 12 000g at 4°C for 10 min. Supernatant protein concentrations were measured by the BCA method on a microplate reader. The protein extracts were separated by sodium dodecyl sulfate polyacrylamide gel electrophoresis and wet transferred to PVDF immobilon P membranes (Bio Rad). The membranes were blocked with 5% non-fat milk for 1.5h at room temperature and then incubated with primary antibodies overnight at 4°C (NRG1 antibody: ab32375, Abcam; ErbB4 antibody: ab180808 Abcam). After primary antibody incubation, membranes were washed with TBST buffer. Secondary antibody dilutions were added and incubated for 1 h at room temperature. Antibody binding was detected using an enhanced chemiluminescence detection system. The bands were analyzed using Image J software.

### Statistics

The results for physiological measurements were presented as the mean ± standard deviation of the mean (SD). Analyses of differences among groups were measured by one-way analysis of variance (ANOVA) with SPSS26.0. The software GraphPad Prism 8.0.1 was used for statistical analyses. A significant level was set at a p-value < 0.05.

## Results

### Blood Glucose and Insulin Resistance

The body weights of diabetic rats (D, DE, and DM) were significantly lower than those of control (C) rats (all p<0.05), with no significant differences in body weights in group D, DE, and DM (all p>0.05). Fasting glucose levels were reduced in groups DE and DM groups compared with diabetic (D) (p<0.01), indicating that both interventions alleviated hyperglycemia. Furthermore, insulin levels in groups D, DE and DM were decreased compared with rats in group C (all p<0.05), with no differences detected in the three groups of diabetic rats (all p>0.05).

**Figure 1:**
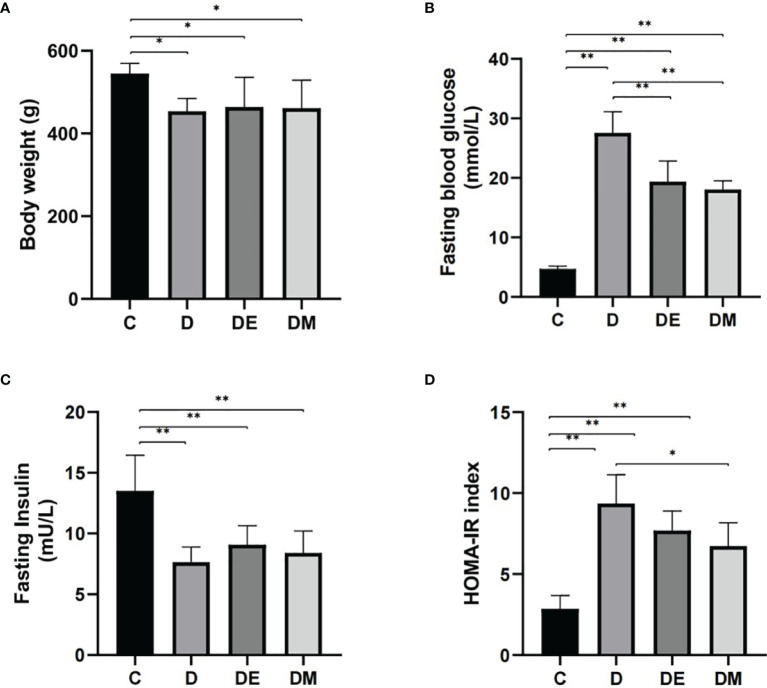
Body weights, fasting blood glucose, fasting insulin, and HOMA-IR after 8 weeks of aerobic exercise and MOTS-c treatment. **(A)** Body weights were higher in rats from group DE than in group DM (p < 0.05). **(B)** Values for FBG in DE and DM were significantly lower than in group D (p < 0.01). **(C)** Rats from groups D, DE and DM groups had lower FINS compared with rats from group C (p < 0.01). **(D)** Values for HOMA-IR in group DM were significantly lower than in group D (p < 0.05). n = 10 rats per group; *p < 0.05, **p < 0.01; C (control), D (diabetic), DE (diabetic+ exercise), DM (diabetic + MOTS-c).

Insulin resistance was determined by HOMA-IR as calculated using fasting glucose and fasting insulin levels. Rats in group D had a higher HOMA-IR (p<0.01), indicating insulin resistance. The HOMA-IR index of rats in group DM were decreased compared with group D (p<0.05). HOMA-IR of rats in DE tended to be lower, there were no significant differences between groups DE and D (p>0.05) (**Figure 1**).

### Cardiac Structure and Function

#### Myocardial Morphological Changes

**Figure 2:**
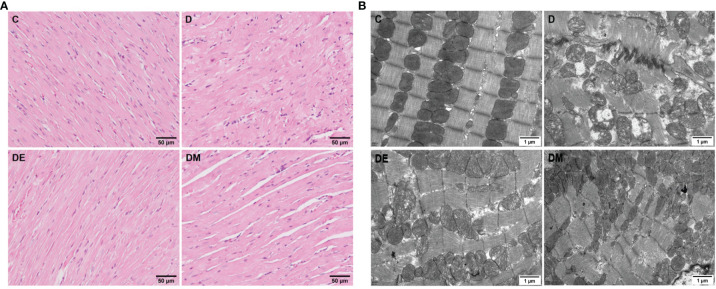
Myocardial morphological changes after 8 weeks of aerobic exercise and MOTS-c treatment. **(A)** HE staining images of left ventricular sections. **(B)** Cardiac ultra-structure detected using transmission electron micrograph. C (control), D (diabetic), DE (diabetic+ exercise), DM (diabetic + MOTS-c).

Rats from group D had cardiac atrophy, derangement of muscle fibers, and partial fibrinolysis; the micro vessels appeared to be damaged and micro bleeding. Rats in groups DE and DM underwent myocardial thinning, although microvascular damage and microbleeds were also observed (**Figure 2A**). Images from TEM showed swollen mitochondria that were vacuolated in group D, with broken cristae. The structure of mitochondrial cristae in diabetic rats treated with exercise and MOTS-c were restored to normal without signs of mitochondrial vacuolization (**Figure 2B**). These observations indicated that the extent of myocardial injury in the groups DE and DM was lower than that in group D.

#### Cardiac Diastolic and Systolic Function Changes

**Figure 3:**
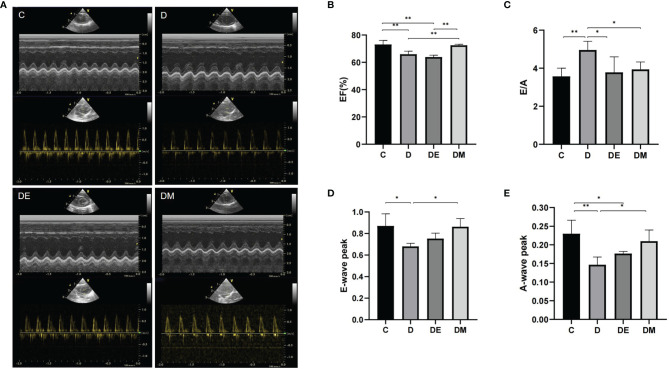
Cardiac systolic and diastolic function after 8 weeks of aerobic exercise and MOTS-c treatment. **(A)** M-mode echocardiographic images and echocardiographic Doppler color flow images **(B)** Rats from groups D, DE had lower EF values compared with rats from groups C (p < 0.01) or DM (p < 0.01), with no significant differences in EF between rats from groups DM and C (p > 0.05). **(C)** The E/A ratio in rats from groups DE and DM were decreased compared with rats from group D (both p < 0.05), but was similar with those in group C (p > 0.05). **(D)** Rats from group DE had lower E-wave peak values compared with DM group (p < 0.05). **(E)** Values of A-wave peak in group DM were higher than that in group D (p < 0.05), with no differences between DM and C (p > 0.05). n = 10 rats in each group; *p < 0.05, **p < 0.01; C (control), D (diabetic), DE (diabetic+ exercise), DM (diabetic + MOTS-c).

Images from M-mode echocardiography are shown in **Figure 3**. Values of EF in group D were lower than that in group C (p<0.01). Peak E-waves and A-waves in group D were decreased compared to group C (p<0.05, p<0.01 respectively), with more rapid decreases of the A-leading to increases in the E/A ratio. Values of E-wave peaks and E/A ratios from group DE were not different from those in group C (p>0.05), while A-wave peak was decreased compared with group C (p<0.05), but still higher than group D (p<0.05). EF was not increased (p>0.05) and remained similar to values in group D, indicating that exercise increased the diastolic function in diabetic rats without changing systolic function. There was no difference in other functional indices in groups DM and C (all p>0.05). All indices of cardiac function in group DM were improved compared to group D (all p>0.05).

### Identification of Differentially Expressed Genes (DEGs)

**Figure 4:**
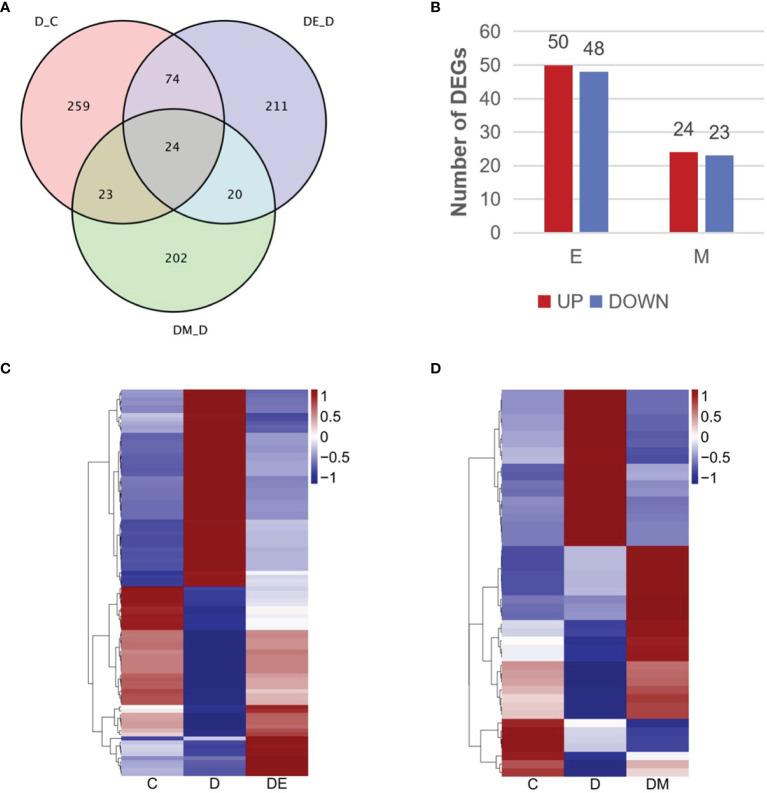
Transcriptomic profiling of differentially expressed genes. **(A)** Venn diagram describing the overlap of DEGs in the three comparisons (D vs. C, DE vs. D, DM vs. D). **(B)** Numbers of upregulated and downregulated DEGs in the three comparisons. **(C, D)** Heatmap representing the relative expression levels of DEGs in the three comparisons. Blue and red represent down and up regulation respectively, and white shows no significant change. C (control), D (diabetic), DE (diabetic+ exercise), DM (diabetic + MOTS-c), E (D-C vs. DE-D), M (D-C vs. DM-D).

We analyzed the transcriptomic profiling of differentially expressed genes to explore the molecular mechanisms of aerobic exercise and MOTS-c induced cardio protection in diabetes. A comparison of D vs. C (D-C) identified genes involved in the pathogenesis of diabetes, while comparing DE vs. D (DE-D) measured DEGs in diabetic rats after 8 weeks of aerobic exercise. A comparison of overlapping DEGs in the comparison of D-C vs. DE-D (E) indicated the genes altered by exercise intervention, and likewise, changes in DEGs produced by MOTS-c were identified by the comparison of D-C vs. DM-D (M). The quantitative analysis of DEGs was summarized in the Venn diagram illustrated in **Figure 4A**.

We identified 121 DEGs in the comparisons of E and M (**Figure 4A**), and 98 genes from a comparison of D-C vs. DE-D, where 50 genes were upregulated and 48 were downregulated (**Figure 4B**). MOTS-c treatment for 8 weeks led to 47 DEG as shown in the comparison of D-C vs. DM-D, of which 24 genes were upregulated, and 23 genes were downregulated (**Figure 4B**). We then analyzed the expression patterns of all DEGs using hierarchical clustering and illustrated the findings in heatmaps (**Figures 4C, D**). The gene expression in group D tended to be the opposite as in group C, while gene expression of groups DE and DM were similar to that in group C.

### Functional Enrichment Analysis of DEGs

To explore the potential functions of DEGs and related pathways of MOTS-c and exercise induced improvements in diabetic cardiac dysfunction, we performed gene ontology (GO) enrichment analysis by GlueGO plug-in of Cytoscape. The DEGs were divided into three ontologies: biological processes (BP), cellular compartments (CC), and molecular functions (MF).

**Figure 5:**
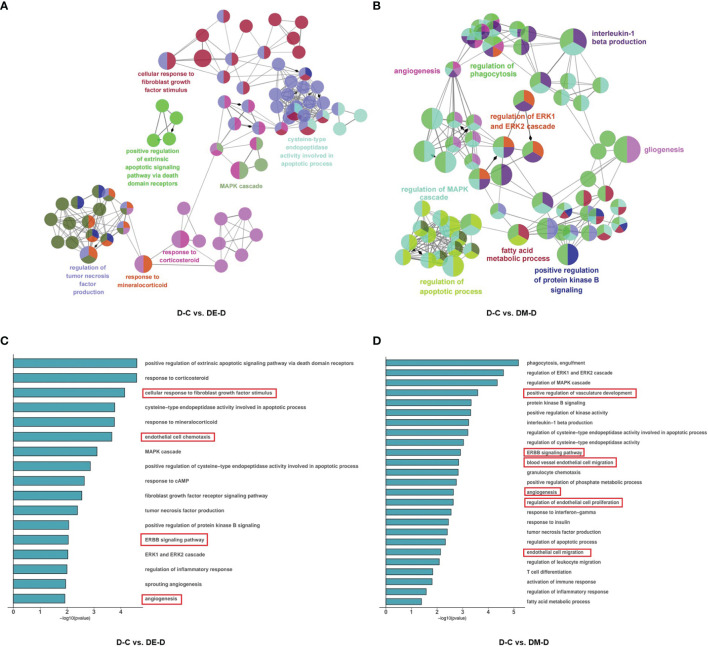
Functional enrichment analysis of DEGs. **(A, B)** Network based analysis of significantly enriched GO terms when comparing DE-D vs. D-C and DM-D vs. D-C. **(C, D)** Histograms with highly enriched GO terms related to myocardium. A red border indicates terms related to endothelial cell and angiogenesis. C (control), D (diabetic), DE (diabetic+ exercise), DM (diabetic + MOTS-c).

The 98 DEGs identified when comparing D-C vs. DE-D were enriched in 98 GO terms and distributed as 96 for BP, 2 for CC and 0 for MF. These GO terms were further clustered into seven groups and visualized using ClueGO (**Figures 5A, B**), and included the mitogen-activated protein kinase (MAPK) cascade, responses to corticosteroids, fibroblast growth factor, mineralocorticoids, regulation of tumor necrosis factor (TNF) production, cysteine-type endopeptidase activity involved in apoptotic processes, and positive regulation of extrinsic apoptotic signaling pathways *via* death domain receptors. The results suggest that angiogenesis, endothelial cell chemotaxis and ErbB signaling pathways can improve vasodilation, and which can improve cardiac diastolic function (**Figure 5C**).

In the comparison of DM-D vs. D-C, 47 DEGs were enriched at significant levels in 195 GO terms, of which 188 were enriched in BP, 2 were enriched in CC, and 5 were enriched in MF. Function enrichment analysis showed that the GO terms were mainly related to angiogenesis, regulation of the apoptotic process, regulation of MAPK cascade, positive regulation of protein kinase activity, fatty acid metabolic process, regulation of phagocytosis, positive regulation of protein kinase B signaling, regulation of ERK1/2 (extracellular regulated protein kinases) cascade, gliogenesis, and interleukin-1 beta production (**Figure 5B**), with highly significant enrichment of pathways for angiogenesis, endothelial cell proliferation and ErbB, similar to findings after aerobic exercise (**Figure 5D**).

### The Effects of Exercise and MOTS-c on NRG1-ErbB Signaling

The ErbB signaling pathway was enriched when comparing DE-D vs. D-C and DM-D vs. D-C, where neuregulin1(NRG1) is a component of the ErbB pathway. ErbB is a receptor for NRG1, a cardiomyocyte mitogen that increases myocardial angiogenesis in rats with diabetic cardiomyopathy (42). The NRG1/ErbB pathway also induces cardiomyocyte proliferation and regulates angiogenesis and vascular endothelial function (43–45).

NRG1-ErbB4-C/EBPβ signaling is a key pathway implicated in regulating the adaptation in cardiomyocytes induced by exercise (46, 47). Our previous findings suggested that both MOTS-c and aerobic exercise improved myocardial performance in rats (48). Based on the results of function enrichment analysis of DEGs and the exercise-like effects of MOTS-c in myocardium, we determined the mRNA and protein expression of NRG1 and ErbB4 to explore the molecular mechanisms of MOTS-c induced beneficial effects in cardiac.

**Figure 6:**
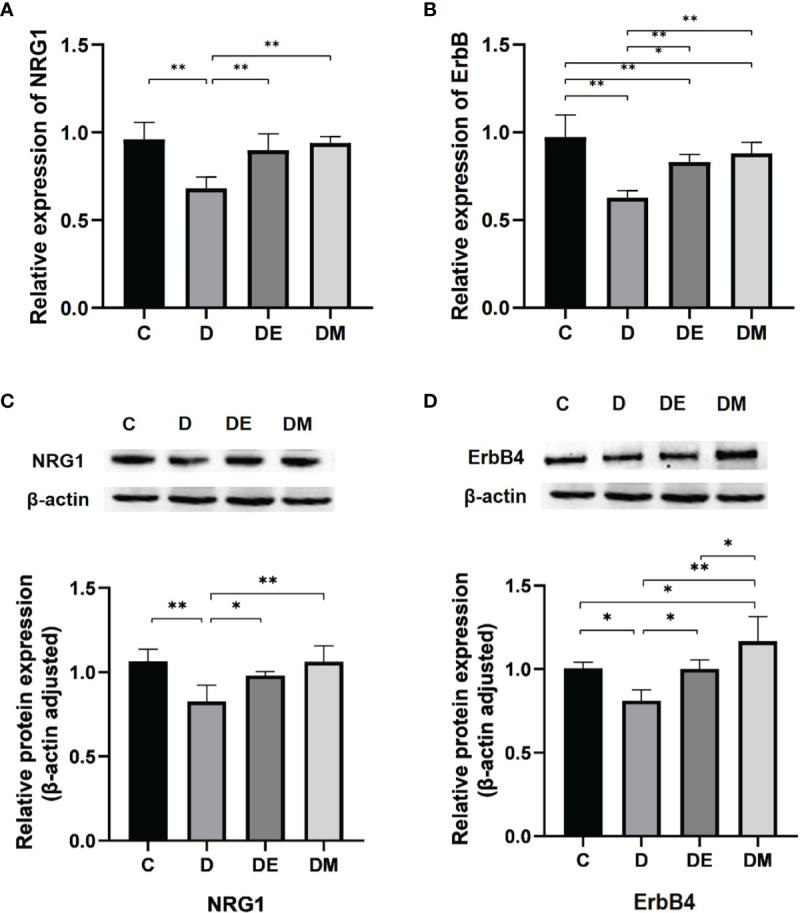
The mRNA expressions of NRG1 and ErbB. **(A, B)** Rats from group D had lower NRG1 and ErbB mRNA expression compared with groups C, DE and DM (all p < 0.01), while the ErbB mRNA expression of rats in group C was higher than groups DE (p < 0.01) and DM (p < 0.05). **(C, D)** The protein levels of NRG1 (p < 0.01) and ErbB4 (p < 0.05) were significantly lower in group D than group C NRG1 and ErbB protein levels were significantly higher in group DE (both p < 0.05 and very significantly higher in group DM (both p < 0.01) than those in group D n = 10 rats per group; *p < 0.05, **p < 0.01; C (control), D (diabetic), DE (diabetic + exercise), DM (diabetic + MOTS-c).

Rats from group D had lower NRG1 and ErbB mRNA expression compared with those from groups C, DE and DM (all p<0.01). The protein levels of NRG1 (p<0.01) and ErbB4 (p<0.05) were significantly lower in group D than C, which is consistent with the results of mRNA expression. NRG1 mRNA expression of group C has no significant difference with that in groups DE and DM (both p>0.05). ErbB mRNA expression in group C was significantly higher than that in groups DE (p<0.01) and DM (p<0.05). Meanwhile, the protein levels of NRG1 and ErbB were significantly higher in group DE (both p<0.05) and very significantly higher in group DM (both p<0.01) than those in group D. (**Figure 6**).

## Discussion

Diabetes mellitus (DM) is a chronic disease characterized by hyperglycaemia (49) and has long-term complications that include peripheral vascular disease, retinopathy, neuropathy, and eventually, cardiac dysfunction/failure or cardiomyopathy (50), and diastolic dysfunction of varying degrees (51–53). We reported that 8 weeks aerobic exercise and administration of exogenous MOTS-c lowered blood glucose and insulin resistance in diabetic rats, while also improving the structures of myocardial fibres and mitochondria. In addition, EF in diabetic control rats was reduced, while E/A was increased due to decreases in the A-wave peak, suggesting impaired diastolic and systolic function in diabetic rats. It is generally recognized that decreases in the E peak leads to compensation of atrial contraction during diastolic dysfunction, causing increases in the A peak and a lowering of the E/A ratio (54, 55), although our results for the E/A ratios are not in agreement with other findings (56, 57). We measured greater decreases in the A peak than E peak in diabetic rats, resulting in an increased E/A ratio, which may be caused by impaired cardiac systolic function in diabetic rats. All indicators were improved (to different levels) after exercise and MOTS-c treatment, with MOTS-c having superior effects on both systolic and diastolic function.

Exercise decreases the risk of type 2 diabetes, myocardial and vascular disease, and all-cause mortality (58, 59). It is unknown if MOTS-c, by acting as an exercise mimetic, has similar benefits in improving diabetic heart disease. Our transcriptome analysis indicated that 8 weeks aerobic exercise and MOTS-c treatment altered 98 and 47 pathogenic genes, respectively, of which 24 genes overlapped, while functional enrichment analysis identified similarity between the effects of MOTS-c and exercise on angiogenesis, inflammation and apoptosis. These findings suggest both MOTS-c and exercise mitigate diabetic cardiac dysfunction *via* similar mechanisms.

Microangiopathy is an important cause of diabetic cardiomyopathy. Hyperglycaemia-induced microvascular changes are manifested as endothelial cell dysfunction (60). The GO analysis in our study showed that both exercise and MOTS-c treatments increased the expression of genes related to vascular endothelial cell proliferation and angiogenesis, a finding that is supported by a meta-analysis indicating that aerobic exercise mitigates endothelial dysfunction in type 2 diabetes (61). Exercise enhances shear stresses on the endothelium, leading to increased nitric oxide (NO) synthesis and vasodilation (62, 63), while also restoring the function of endothelial progenitor cells, promoting endothelial repair and facilitating vascular angiogenesis (64). Serum levels of MOTS-c are significantly reduced in subjects with type 2 diabetes (65). Low plasma levels of MOTS-c occur in patients with endothelial dysfunction, where there is a positive correlation between plasma MOTS-c levels and coronary microvascular endothelial function (66). Treatment with MOTS-c improves endothelial function in a model of renovascular hypertension due to renal artery stenosis (67).

**Figure 7:**
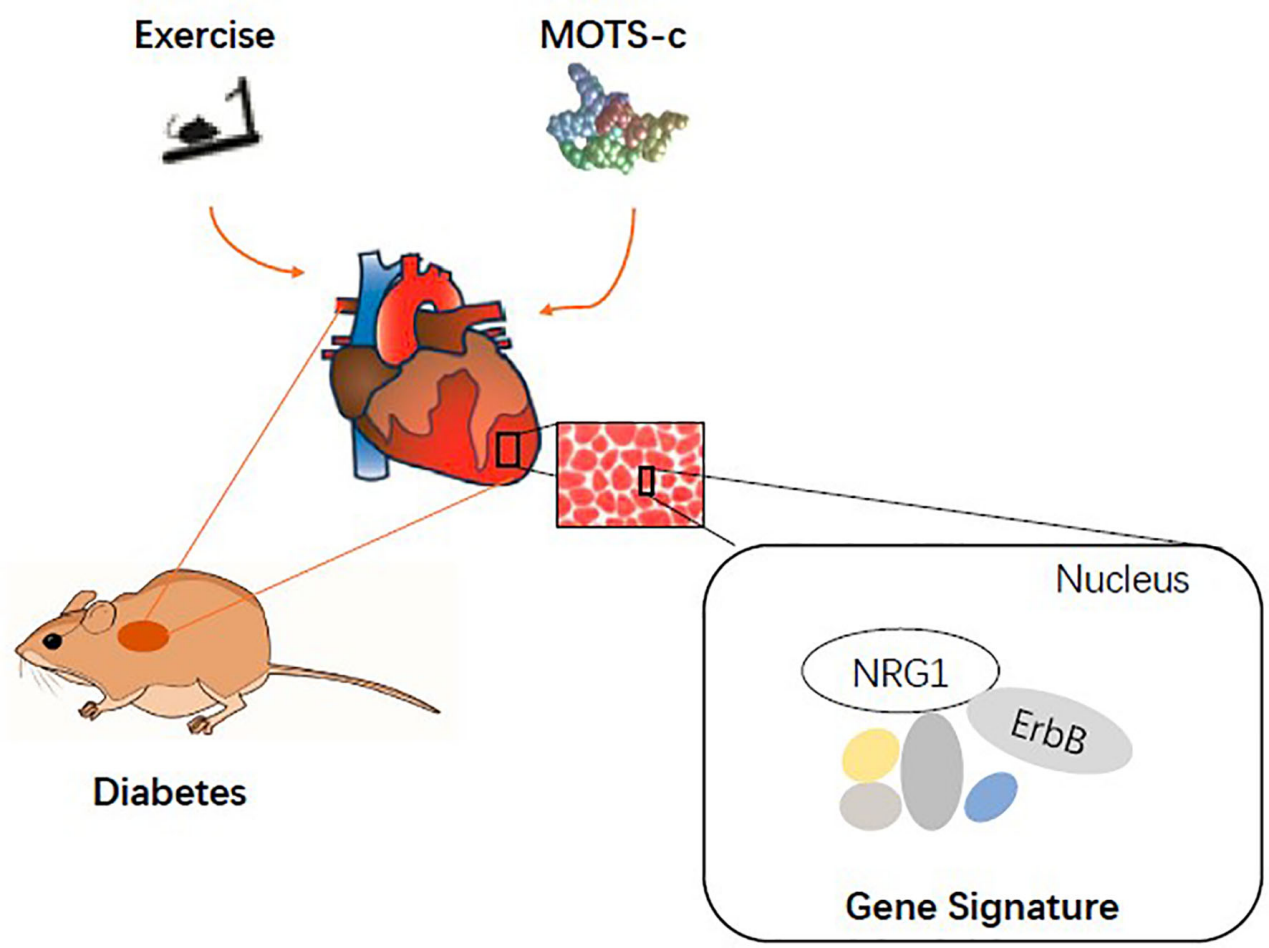
Model depicting the overlapping mechanisms between exercise and MOTS-c supplements in restoring cardiac function in diabetes.

Importantly, GO analysis indicated both exercise and MOTS-c enriched the ErbB signalling pathway involving the NRG1 gene. Endothelial cells express ErbB receptors that are targets for autocrine signalling *via* the NRG1/ErbB pathway, which mediates vascular integrity and angiogenic responses of the endothelium (44). The expression of cardiac NRG1 and the phosphorylation of ErbB2 and ErbB4 are reduced in *in vitro* (cellular) and *in vivo* (rat models) of diabetic cardiomyopathy (68, 69). Serum levels of NRG-1β protein are lower in patients with heart failure (70). We reported decreased expression of NRG1 mRNA levels in the diabetic myocardium, which was restored to near normal levels by aerobic exercise and MOTS-c. Untreated diabetic rats had a lower expression of ErbB mRNA compared to those treated with aerobic exercise and MOTS-c. Aerobic exercise and MOTS-c treatment upregulate NRG1 and ErbB mRNA expression to restore the decreased NRG1/ErbB signalling pathway in diabetes. Our data are supported by the findings of Cai et al. that exercise training up-regulates NRG1 expression and activates ErbB2, ErbB4 to promote cardiac repair by stimulating endogenous regeneration (71) (**Figure 7**).

## Study Limitations

Firstly, our study is limited to a certain dose of MOTS-c that is based on a previous study (29), and should be expanded to include differences related to concentration and route and frequency of administration. Secondly, we did not investigate the transcriptomic profiling characters of different exercise protocols (such as: moderate vs. vigorous, aerobic vs. resistance) on the diabetic myocardium. Finally, only intergroup different expression genes were involved in the transcriptomic analysis, so creating some limitations when considering screening pathways and key regulatory genes. Further research with aerobic exercise plus MOTS-c intervention to explore the overlapping and additive effects of MOTS-c and exercise will provide additional mechanistic details on diabetic myocardial dysfunction.

## Conclusions

Both aerobic exercise and MOTS-c treatment significantly reduce abnormalities in diabetic cardiac structure and function. Exercise and MOTS-c induced overlapping differentially expressed genes, with function enriched in inflammation, myocardial apoptosis, angiogenesis and endothelial cell proliferation, of which, the NRG1-ErbB4 pathway may be key to the exercise-mimicking effects of MOTS-c on restoring cardiac dysfunction in diabetes. Our study may aid in better understanding of the effects of MOTS-c and aerobic exercise in diabetic cardiac diastolic dysfunction and explore whether exogenous MOTS-c treatment might mimic exercise-induced myocardial transcriptional response in diabetes.

## Data Availability Statement

The datasets presented in this study can be found in online repositories. The names of the repository/repositories and accession number(s) can be found below: National Center for Biotechnology Information (NCBI) BioProject database under accession number PRJNA804364.

## Ethics Statement

The animal study was reviewed and approved by Academic Committee of Chengdu Sport University (No: 2021-07).

## Author Contributions

Conceptualization, SL and IL. Methodology, SL and MW. Software, JM. Validation, XP, YP, and JY. Formal analysis, YF. Data curation, MW and JM. Writing—original draft preparation, SL. Writing—review and editing, IL. Visualization, MW. Supervision, IL. Project administration, SL. Funding acquisition, SL. All authors have read and agreed to the published version of the manuscript.

## Funding

This research was funded by National Natural Science Foundation of China, grant number 31971104.

## Conflict of Interest

The authors declare that the research was conducted in the absence of any commercial or financial relationships that could be construed as a potential conflict of interest.

## Publisher’s Note

All claims expressed in this article are solely those of the authors and do not necessarily represent those of their affiliated organizations, or those of the publisher, the editors and the reviewers. Any product that may be evaluated in this article, or claim that may be made by its manufacturer, is not guaranteed or endorsed by the publisher.
